# Sodium channel Na_v_1.6 accumulates at the site of infraorbital nerve injury

**DOI:** 10.1186/1471-2202-8-56

**Published:** 2007-07-27

**Authors:** Michael A Henry, Angelique R Freking, Lonnie R Johnson, S Rock Levinson

**Affiliations:** 1 Department of Endodontics, University of Texas Health Science Center at San Antonio, San Antonio, TX 78229, USA; 2 Department of Physiology and Biophysics, University of Colorado at Denver and Health Sciences Center, Aurora, CO 80045, USA; 3 Department of Surgical Dentistry, University of Colorado at Denver and Health Sciences Center, Aurora, CO 80045, USA

## Abstract

**Background:**

Sodium channel (NaCh) expressions change following nerve and inflammatory lesions and this change may contribute to the activation of pain pathways. In a previous study we found a dramatic increase in the size and density of NaCh accumulations, and a remodeling of NaChs at intact and altered myelinated sites at a location just proximal to a combined partial axotomy and chromic suture lesion of the rat infraorbital nerve (ION) with the use of an antibody that identifies all NaCh isoforms. Here we evaluate the contribution of the major nodal NaCh isoform, Na_v_1.6, to this remodeling of NaChs following the same lesion. Sections of the ION from normal and ION lesioned subjects were double-stained with antibodies against Na_v_1.6 and caspr (contactin-associated protein; a paranodal protein to identify nodes of Ranvier) and then z-series of optically sectioned images were captured with a confocal microscope. ImageJ (NIH) software was used to quantify the average size and density of Na_v_1.6 accumulations, while additional single fiber analyses measured the axial length of the nodal gap, and the immunofluorescence intensity of Na_v_1.6 in nodes and of caspr in the paranodal region.

**Results:**

The findings showed a significant increase in the average size and density of Na_v_1.6 accumulations in lesioned IONs when compared to normal IONs. The results of the single fiber analyses in caspr-identified typical nodes showed an increased axial length of the nodal gap, an increased immunofluorescence intensity of nodal Na_v_1.6 and a decreased immunofluorescence intensity of paranodal caspr in lesioned IONs when compared to normal IONs. In the lesioned IONs, Na_v_1.6 accumulations were also seen in association with altered caspr-relationships, such as heminodes.

**Conclusion:**

The results of the present study identify Na_v_1.6 as one isoform involved in the augmentation and remodeling of NaChs at nodal sites following a combined partial axotomy and chromic suture ION lesion. The augmentation of Na_v_1.6 may result from an alteration in axon-Schwann cell signaling mechanisms as suggested by changes in caspr expression. The changes identified in this study suggest that the participation of Na_v_1.6 should be considered when examining changes in the excitability of myelinated axons in neuropathic pain models.

## Background

Voltage-gated sodium channels (NaChs) are recognized as a diverse group that consist of at least nine different subtypes or isoforms [1]. The activation of NaChs is a key event leading to action potential generation and impulse propagation [2]. These isoforms are differentially distributed throughout the nervous system and show important changes in expression after inflammatory and axotomy insults and some of these changes may contribute to the development and maintenance of pain states [3]. Much attention has been placed on the evaluation of changes in the expression of specific isoforms after lesions and especially of those that are preferentially expressed in the peripheral nervous system [4]. Much less is known about changes in expression after peripheral injury of isoforms that are more widely expressed in both the peripheral and central nervous systems, such as Na_v_1.6.

The Na_v_1.6 isoform is strongly expressed by sensory neurons [5], located in unmyelinated fibers [6] and also represents the isoform located at nodes of Ranvier [5,7]. The node of Ranvier contains a high density of NaChs whose activation is necessary for saltatory conduction [8] and thus represents a key region influencing the excitability of myelinated fibers. Changes in the density or distribution of NaChs at the node of Ranvier may contribute to changes in excitability that follow experimental nerve insults or in disease states. Even though Na_v_1.6 plays a key role in the propagation of action potentials throughout the nervous system, studies that have evaluated changes in its expression in pain states are limited [9].

We are studying the role of altered NaCh expression in trigeminal pain states and have used a combined partial axotomy and chromic suture lesion of the rat infraorbital nerve (ION) that produces a behavior characterized by increased sensitivity to mechanical stimuli as a model system where we can quantify changes in expression within single fibers [10]. Through the use of this model and methodology, we have described significant remodeling and augmentation of NaCh immunofluorescence within intact and presumably demyelinating nodes of Ranvier with the use of a "pan-specific" antibody that recognizes a conserved sequence seen in the alpha subunit of all vertebrate NaCh isoforms [11,12]. In this study we use the same lesion and evaluate the contribution of the Na_v_1.6 isoform to the remodeling of NaChs that was identified with the pan-specific antibody used in the earlier study.

## Results

### Behavioral response to monofilament stimulation

Monofilament stimulation of the vibrissa pad two weeks after the ION lesion showed less force was needed to produce a threshold behavioral response (defined as head withdrawal, bite filament, attack filament, prolonged directed grooming, and/or scratching of the stimulated vibrissa pad) on the side of the lesion when compared to that seen in normal control subjects. The four normal control subjects showed either a lack of threshold response or threshold response only with the maximum 26 gram force stimulus, while the experimental subjects individually showed threshold responses after 1, 2, 6 and 15 gram force stimulations.

### Qualitative observations of Na_v_1.6 and caspr staining patterns and relationships in normal vs. lesioned IONs

**Figure 1:**
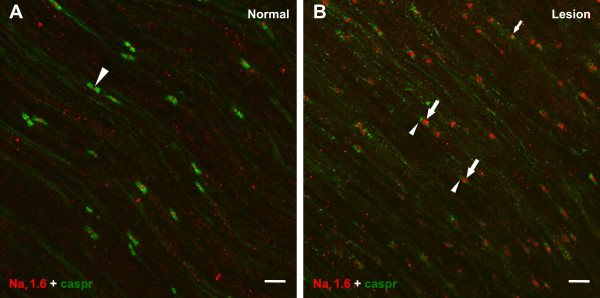
**Na_v_1.6 and caspr staining**. **A **and **B**. Confocal micrographs from average-intensity z-projections (45 slices in A; 47 slices in B) demonstrate Na_v_1.6 (red) and caspr (green) staining relationships seen within the normal (A) and lesioned (B) ION. **A**. The normal ION shows bright Na_v_1.6 staining (arrowhead) at nodes of Ranvier that are identified as the gap seen between the paranodal staining of caspr. The caspr staining is intense within the paranodal region and prominent Na_v_1.6 staining is generally restricted to the narrow area at the node. **B**. The lesioned ION, at a location just proximal to the chromic suture, shows a change in the Na_v_1.6 and caspr staining relationships that were seen in A. These changes include a loss in the overall organization and the immunofluorescence intensity of caspr staining within the paranodal region (arrowheads) and an increased size and intensity of Na_v_1.6 within accumulations (large arrows). Alterations in the normal Na_v_1.6 and caspr staining relationships include Na_v_1.6 accumulations that show an association with caspr on one side (heminodes; small arrow). Both images were collected with the same laser settings. Scale bars = 10 μm.

**Figure 2:**
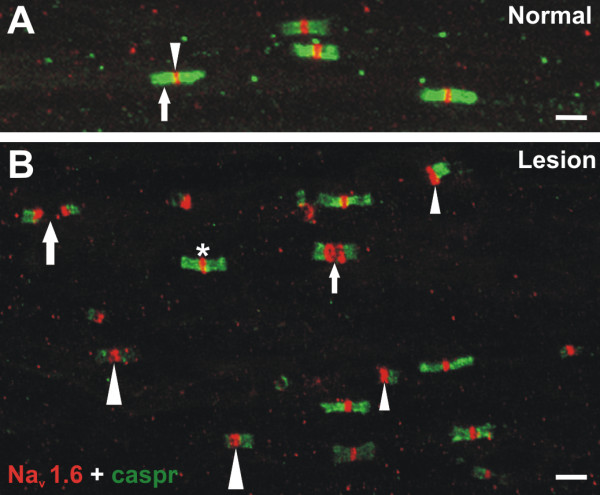
**Na_v_1.6 and caspr staining**. **A **and **B**. Confocal migrographs of Na_v_1.6 (red) and caspr (green) staining relationships seen within individual fibers in the normal (A) and lesioned (B) ION. **A**. Individual fibers within the normal ION show bright Na_v_1.6 staining (arrowhead) at nodes of Ranvier that are identified as the gap seen between the dense paranodal staining of caspr (arrow). **B**. Individual fibers within the lesioned ION show alterations in the Na_v_1.6 and caspr staining relationships that include "heminodes" (small arrowheads) and "split nodes" that vary in the distance seen between the Na_v_1.6 accumulations (small and large arrows). Some large Na_v_1.6 accumulations are flanked on both sides by reduced caspr staining (large arrowheads), while other nodes appear more normal (asterick). Scale bars = 5 μm.

Examination of the Na_v_1.6 and caspr-staining patterns and relationships with the confocal microscope revealed a dramatic difference in the extent and intensity of immunofluorescence of both Na_v_1.6 and caspr between normal and lesioned IONs (Figs. 1 and 2). Typical nodes of Ranvier were identified as the gap seen between the paranodal staining of caspr with the Na_v_1.6 staining at nodes in normal IONs assuming a prominent disc-like appearance (Figs. 1A and 2A). The caspr staining was typically intense and selective within the paranodal region and associated with sharp demarcation borders. Most of the large Na_v_1.6 accumulations with bright staining intensity were associated with prominent caspr staining seen in the paranodal regions. Weak staining of caspr was also seen along some nerve fibers.

In contrast, the typical association of intense Na_v_1.6 at the node bordered on both sides by bright caspr staining in the paranodal area was altered in lesioned IONs (compare Fig. 1A to 1B and Fig. 2A to 2B). In general, both the intensity and the area occupied by Na_v_1.6 immunofluorescence increased at nodes, while the paranodal staining of caspr appeared attenuated. The Na_v_1.6 accumulations in lesioned tissue appeared larger, brighter and more ovoid rather than the disc-like appearance seen in normal IONs. The caspr staining associated with these "typical" nodes also appeared less intense. The common relationship of caspr with Na_v_1.6 was also altered in some accumulations and included the presence of "heminodes" where caspr staining was located on only one side of a contiguous Na_v_1.6 accumulation and "split nodes" where two distinct NaCh accumulations were separated by a gap in the same fiber and with each accumulation flanked on one side by caspr (Fig. 2B). Finally, the total number of Na_v_1.6 accumulations appeared to be significantly increased in the lesioned tissue (i.e. density appeared to be much higher). In contrast to the dense Na_v_1.6 staining seen at nodes, selective labeling of fibers that lacked an association with caspr, and that most likely represent unmyelinated fibers, was absent in the IONs of both control and lesioned subjects.

Since the apparent density, size (including the nodal gap axial length) and intensity of Na_v_1.6 accumulations, and the intensity of caspr staining in the paranodal region of typical nodes appeared so different in lesioned IONs when compared to normal IONs, these observations were evaluated further using quantitative techniques.

### Quantitative evaluation of the density and size of Na_v_1.6 accumulations in normal vs. lesioned IONs

**Figure 3:**
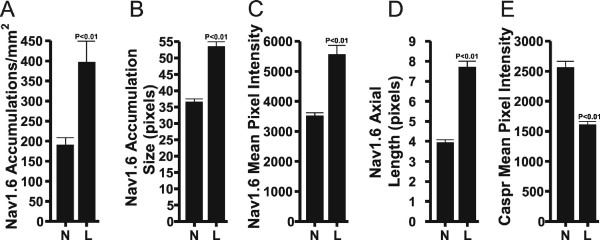
**Results of quantitative analyses performed in normal (N) and lesioned (L) infraorbital nerves**. **A **and **B**. The density (A) and average pixel size (B) of Na_v_1.6 accumulations (with a size of ≥ 15 pixels) was significantly greater in lesioned IONs than seen in normal IONs. **C**. The average immunofluorescence intensity (0–65,535 range) of pixels with Na_v_1.6 staining located in caspr-identified typical nodes was significantly greater in lesioned IONs when compared to normal IONs. **D**. The average axial length (in pixels) of the nodal gap within caspr-identified typical nodal accumulations was significantly greater in lesioned IONs when compared to normal IONs. **E**. The average immunofluorescence intensity (0–65,535 range) of pixels with caspr staining located within the paranodal regions of caspr-identified typical nodes was significantly greater in normal IONs when compared to lesioned IONs.

Quantitative evaluation determined that the density (Fig. 3A) and size (Fig. 3B) of Na_v_1.6 accumulations were both significantly greater in the lesioned IONs when compared to normal IONs. The density analysis (as determined by the number of Na_v_1.6 accumulations/mm^2^) calculated a density of 397.6 +/- 51.6 (SEM) accumulations/mm^2 ^in lesioned IONs and 191.2 +/- 17.4 (SEM) accumulations/mm^2 ^in normal IONs (p < 0.01 significance level; Fig. 3A). The average size (in pixels) of Na_v_1.6 accumulations was 53.6 +/- 1.4 (SEM) in lesioned IONs and 36.6 +/- 0.86 (SEM) in normal IONs (p < 0.01 significance level; Fig. 3B).

### Quantitative evaluation of Na_v_1.6 and caspr immunofluorescence staining intensities and nodal gap axial length within single caspr-identified typical nodes in normal vs. lesioned IONs

The average intensity of pixels with Na_v_1.6 immunofluorescence located within typical nodes was significantly greater (p < 0.01) in lesioned IONs when compared to normal IONs (5571.8 +/- 294.1 (SEM) vs. 3529.6 +/- 90.3 (SEM); Fig. 3C). In addition, the analysis of typical nodesalso showed that the average nodal gap axial length as measured by the number of pixels in the caspr-demarcated gap that is occupied by Na_v_1.6, was significantly greater (p < 0.01) in lesioned IONs than in normal IONs (7.72 +/- 0.29 (SEM) vs. 3.95 +/- 0.13 (SEM); Fig. 3D). Thus Na_v_1.6 nodal clusters appear to have significantly broadened in lesioned IONs. In contrast, the evaluation of the average intensity of pixels with caspr immunofluorescence within the paranodal region of typical nodes showed that this intensity was significantly less (p < 0.01) in the lesioned IONs when compared to normal IONs (1614.7 +/- 48.2 (SEM) vs. 2565.4 +/- 102.1 (SEM); Fig. 3E).

## Discussion

In a previous study we described striking changes in NaCh expression in a location just proximal to a combined partial axotomy and chromic suture lesion of the ION [10]. The present investigation specifically identifies the involvement of the Na_v_1.6 isoform in the augmentation and remodeling of NaChs at intact and presumptive demyelinating sites and in the increased density of NaCh accumulations that were previously identified with the use of the pan-specific NaCh antibody in this nerve injury model. Most studies that have evaluated the role of altered NaCh expression in peripheral pain states (inflammatory and/or neuropathic) have focused on the role of the Na_v_1.7, 1.8 and 1.9 isoforms that are preferentially expressed in the peripheral nervous system and seen in a subset of nociceptors [13-15] and on the re-expression of Na_v_1.3 after injury [16]. Many of these have evaluated changes in protein and gene expressions at the cell body level, while fewer have evaluated changes in single fibers at the site of nerve injury. Although Na_v_1.6 has been identified as the major isoform located at nodes of Ranvier in both the peripheral and central nervous systems [5,7], few studies have examined changes in its localization or expression in disease and injury states in the peripheral nervous system. One study did evaluate Na_v_1.6 expression in an experimental painful diabetic neuropathy rat model and found increased mRNA and protein levels in sensory neurons thus implicating the involvement of this isoform in the generation of altered neuronal activity in this pain condition [9].

Two primary questions that arise from the present observations concern; 1) the mechanisms underlying such changes in sodium channel expression, and 2) the possible role of such changes in the establishment and maintenance of a chronic hyperalgesic state. With respect to the first question, Bennett and Xie introduced the use of chromic suture ligation of the sciatic nerve to produce a rat model of neuropathic pain [17] and this lesion was later adapted to the rat ION [18]. Chromic suture nerve lesions result in behavior that is suggestive of neuropathic pain and this behavior may be due in part to chemicals present in the chromic suture [19]. However, chromic suture produces a potent inflammatory response [20], and this localized inflammatory response may contribute to the development of a demyelinating lesion.

A number of previous studies have shown that disruption of myelin results in profound changes in NaCh expression in axons [10,11,21-26]. Paranodal and segmental demyelination are both associated with a widening of the nodal gap [27] and an augmentation of NaChs can occur within this gap [28], thus accounting for similar observations seen in the present study. This widened nodal gap may result from a disruption of axoglial signaling mechanisms that are critical to the localization of ion channels into the nodal and paranodal regions of the axolemma [29-31]. The reduction of caspr staining seen in the paranodal region within the lesioned IONs has also been seen in axons within demyelinating lesions in patients with multiple sclerosis [32], and most likely reflects altered signaling mechanisms occurring in early stages of paranodal demyelination. Furthermore, NaCh clusters have been reported to form spontaneously in chronically demyelinated axonal segments [22-26], and this could account for the increased number of Na_v_1.6 clusters observed in the present study, particularly those associated with altered caspr relationships such as heminodes. Heminodes are normally seen during development [33] and after experimentally-induced demyelination with lysolecithin [11,27,34,35]. Although it seems likely that most of the changes that we observed after the use of the combined partial axotomy/chromic suture lesion result from alterations in myelination secondary to the use of the chromic suture, our methods did not allow a differentiation of NaCh remodeling in fibers affected by axotomy and chromic suture to those affected by chromic suture alone and so attributes from the axotomy are possible.

The Na_v_1.6 isoform may also be involved in the pathogenesis of demyelination. Studies done in humans and experimental animal models for multiple sclerosis have shown Na_v_1.6 expression in activated glia within the central nervous system [36]. In addition, it has been reported that Na_v_1.6 colocalizes with the Na+/Ca+ exchanger in demyelinated axons with the suggestion that this colocalization may trigger axon injury [21]. Together, these previous findings and our present findings suggest an important role for Na_v_1.6 in both peripheral and central demyelinating conditions.

The role of such pathological changes in NaCh distributions to neuropathic pain mechanisms is less certain. One popular hypothesis is that such localized increases in channel expression results in increased action potential generation near the injury site, thus contributing to the activation of pain pathways [37]. Furthermore, it has been proposed that increased NaCh clusters in demyelinated axons may contribute to repetitive firing and thus act like pacemaker zones that are normally located within axonal endings [38]. Even so, the changes seen here may result in decreased excitability and this possibility identifies the need for additional studies that evaluate changes in NaCh expression with alterations in neuronal excitability.

Since we limited our quantitative evaluation of Na_v_1.6 to those located in large accumulations, such as those found at nodes of Ranvier in myelinated fibers, we most likely did not evaluate possible changes in expression within unmyelinated fibers. Even though Na_v_1.6 has been described in unmyelinated axons (6), the selective labeling of small fibers that lacked paranodal caspr staining was absent in the IONs of both control and lesioned subjects. This difference could be related to the use of different antibodies. Thus, the possible contribution of the Na_v_1.6 accumulations within the myelinated fibers evaluated in the present study to neuropathic pain mechanisms would likely arise through stimulation of the normal A-delta or A-beta pathways. Naturally, possible changes in the expression of Na_v_1.6 in unmyelinated fibers would be important to evaluate in the future since altered activity after inflammatory and axotomy lesions most likely involves contributions from both myelinated and unmyelinated axons.

The development of isoform-specific inhibitors would be useful to further understand the role of each isoform to the development of a complex acute and chronic pain state. Concurrent changes in multiple isoforms are certainly possible and these changes may be site specific at the subcellular level, so that some have greater influence within the axon, soma or central projections of sensory neurons. Nonetheless, evidence is rapidly accruing that sodium channel expression is highly plastic in various forms of inflammatory and neuropathic pain conditions, begging the question of how such changes might be causal to pain mechanisms.

## Conclusion

Our findings identify the involvement of Na_v_1.6 in the augmentation and remodeling of NaChs at nodal sites within single fibers located just proximal to the site of a combined partial axotomy and chromic suture lesion to the ION. The augmentation of Na_v_1.6 may result from an alteration in axon-Schwann cell signaling mechanisms as suggested by changes in caspr expression. The changes identified in this study suggest that the activation of Na_v_1.6 may be important when examining changes in excitability of myelinated axons in various models of inflammatory and neuropathic pain.

## Methods

### Animals and surgical procedures

This study was approved by the University of Colorado Health Sciences Center Animal Care and Use Committee. Four young adult female Sprague-Dawley rats (200–225 grams) were used as experimental subjects, while four were used as normal control subjects in this study. The experimental subjects were anesthetized with an intraperitoneal injection of pentobarbital (10 mgs per subject) or chloral hydrate (80 mgs per subject) and once pain-free the left ION was exposed just distal to the infraorbital foramen by way of a midline incision over the snout. The connective tissue overlying the ION was carefully removed until the multiple fascicles that comprise the nerve were visualized. The ION was released from the deeper tissues by passing a blunt probe under the nerve. Two 4-0 chromic gut sutures (Ethicon Inc., Somerville, New Jersey) were then placed around the ION just distal to the foramen, and the nerve was partially constricted as the sutures were tightened. The lateral half of the ligated nerve fascicles were then transected just distal to the sutures, while the medial half of the nerve fascicles were left intact and thus spared. The superficial incision was closed with Dexon 4-0 suture. Experimental subjects were given acetaminophen in drinking water (2 mg/ml) two days prior to surgery that continued through the fourth day after surgery. Two of the normal control subjects and two of the lesioned subjects were used in a previous study [10].

### Behavioral testing

Experimental and normal control subjects were tested one day before sacrifice for threshold-withdrawal response to graded stimulation of the vibrissae pads with Semmes-Weinstein monofilaments ("Touch-Test Sensory Evaluator": North Coast Medical Inc., Morgan Hill, CA) as previously described [10]. Monofilaments with 1, 2, 4, 6, 8, 10, 15 and 26 gram forces were sequentially applied until a threshold response was obtained (see Results).

### Tissue processing and staining

Following a two week survival period the experimental subjects and normal control subjects were deeply anesthetized with an intraperitoneal injection of pentobarbital (25 mgs) and then transcardially perfused with 100 mls of 0.9% saline in H_2_O followed by 250 mls of fixative consisting of 4% paraformaldehyde in 0.1 M phosphate buffer (PB) at pH 7.4. The ION was exposed and removed extending from its origin at the maxillary/ophthalmic division root to its termination in the vibrissa pad and then post-fixed in the same fixative for 20 minutes at room temperature. The sutures were left in place around the nerves of experimental subjects for orientation purposes. Fixed nerves were rinsed in 0.1 M PB, followed by a rinse in 0.1 M PB with 15% sucrose at room temperature for one hour. The IONs were then placed in 0.1 M PB with 30% sucrose overnight on a shaker at 4°C.

Separate IONs from different subjects were placed side-by-side, embedded in mounting medium (Neg-50; Richard-Allan Scientific, Kalamazoo, MI) and sectioned in the longitudinal plane at 40 μm with the use of a cryostat. Sections were placed onto Superfrost Plus slides (Fisher Scientific, Pittsburgh, PA), allowed to dry and stored in a -20°C freezer. The specimens were removed from the freezer prior to staining and all subsequent steps described below were performed at room temperature. The specimens were rinsed three times in 0.1 M phosphate buffered saline (PBS) for 10 minutes in each rinse. Non-specific binding was decreased by the incubation of the tissue with 2% bovine gamma-globulin (Sigma, St. Louis, MO), 4% normal goat serum (Sigma) and 0.3% Triton X-100 (Fisher Scientific) in 0.1 M PBS for 90 minutes as a blocking solution. The blocking solution was removed and the tissue was incubated in primary antibodies diluted in blocking solution and placed overnight in a humidifier.

An antibody specific to the nodal NaCh isoform Na_v_1.6 [7] was used at a 1:100 concentration, while a caspr (paranodin/contactin-associated protein) monoclonal antibody [39] was used at a 1:500 concentration. The caspr antibody was used to identify nodes of Ranvier that are seen as gaps between the paranodal staining of caspr. The next day the tissue was rinsed in 0.1 M PBS followed by incubation in secondary antibodies diluted to a 1:100 concentration in blocking solution for 90 minutes in a humidifier while protected from the light. An Alexa Fluor 568-conjugated anti-rabbit IgG secondary antibody (red fluorophore; Molecular Probes, Eugene, OR) was used to visualize Na_v_1.6, while an Alexa Fluor 488-conjugated anti-mouse IgG secondary antibody (green fluorophore; Molecular Probes) was used to visualize caspr. Tissues were rinsed in 0.1 M PBS, then water, allowed to dry, coverslipped with Vectashield (Vector Labs, Burlingame, CA) and stored at 4°C. Control tissue specimens were processed as above except the undiluted Na_v_1.6 antibody was preincubated with peptide antigen (200:1 peptide to antibody molar concentration ratio) for a minimum of four hours before the peptide-blocked antibody was diluted and applied to the tissue. Tissue specimens were evaluated and digital immunofluorescence images acquired with a Nikon PCM-2000 laser scanning confocal microscope and SimplePCI software, v4.0 (Compix Inc., Cranberry Township, PA). The evaluation of control tissue specimens showed they lacked Na_v_1.6 staining. The Na_v_1.6 and caspr staining in experimental and control subjects was quantitatively evaluated as described below. Original images were formatted with Adobe Photoshop CS and Corel Photo-Paint 12 for illustration purposes.

### Image acquisition

Longitudinal sections of the IONs that had been double-stained with Na_v_1.6 and caspr antibodies as described above were examined with the confocal microscope. A "z-series" was obtained through each section, at 0.8 μm steps, using a 40X/N.A. 1.30 oil immersion objective. The gain levels used to quantify both Na_v_1.6 and caspr stainings were set by examining tissue from each subject with the confocal microscope to determine the specimen with the brightest pixels. This tissue was then used to determine the laser gain levels where the brightest pixel was just below saturation. These gain levels were then applied for the acquisition of all images. The tissues were minimally evaluated prior to capture of the z-series to avoid photo-bleaching. Z-series images were first obtained for Na_v_1.6-alone staining followed by an additional z-series of the corresponding caspr-alone staining. Similar regions of the nerve in normal and lesioned IONs were selected for imaging and corresponded to the region approximately 0.2 mm proximal to the placement of the most proximal chromic suture in the experimental subjects. Each image was saved as a 16 bit file with a 1024 pixels^2 ^resolution, with each square pixel = 0.3 μm in dimension and representing an area of 0.09 μm^2^.

### Quantification of Na_v_1.6 accumulation density and size

Using NIH ImageJ software [40], matching z-series (Na_v_1.6-alone, caspr-alone, or Na_v_1.6 combined with caspr) were opened simultaneously with each series displayed on its own monitor in a multi-screen array. The combined images allowed characterization of Na_v_1.6 and caspr staining relationships. Quantitative analysis was performed with ImageJ on every 8^th ^slice (6.4 μm separation) within the Na_v_1.6-alone z-series (beginning with the third slice) to determine the average size and density of Na_v_1.6 accumulations within a defined area of the ION. At least four and up to six different slices/images within each Na_v_1.6 z-series were used for this purpose. This distance was used to avoid multiple counts of the same accumulations. The corresponding caspr-alone slice was used to visualize the nerve area of the slice with the aid of the auto button in the "brightness and contrast" menu and this area was outlined with the "freehand selections" tool and added to the "ROI Manager". This was done to exclude areas devoid of fibers. The "Measure" function in the "ROI Manager" was used to determine the total area of the ROI so accumulation density could be determined. The corresponding Na_v_1.6-alone slice was then opened and the image thresholded to a value where low-levels of background staining were mostly eliminated, without the removal of pixels from Na_v_1.6 accumulations. This part of the quantification process was performed twice, and the different threshold values were compared with an unpaired Student's *t*-test. No significant difference was found between these different threshold values. The fiber area ROI was then applied to the thresholded Na_v_1.6 image and the "Analyze Particles" function under the "Analyze" menu was employed to determine the total count and average size of accumulations that were ≥ 15 pixels in size within the ROI.

### Quantification of Na_v_1.6 and caspr immunofluorescence in single fibers

The average staining intensity of nodal Na_v_1.6, the average staining intensity of paranodal caspr, and the axial length of the nodal gap of randomly selected typical nodes were determined in the IONs of the normal and lesioned subjects. This evaluation was limited to typical nodes since the altered types that are commonly seen in lesioned subjects (i.e. heminodes) were rare in normal subjects. Typical nodes were identified and defined by the presence of a dense Na_v_1.6 accumulation flanked on both sides by the paranodal staining of caspr as seen in the Na_v_1.6 combined with caspr z-series. The same typical node was then identified in the Na_v_1.6-alone z-series that was viewed with ImageJ as an average intensity z-projection. The "auto" function in the "brightness and contrast" menu was utilized to better visualize the edges of the pixels of interest within the Na_v_1.6 accumulations. This step allowed an objective method of distinguishing pixels within the Na_v_1.6 accumulation that were included in the evaluation, without altering intensity values. The Na_v_1.6 accumulation was traced with the "freehand" tool and a histogram was generated to collect the number of pixels, average intensity, and standard deviation values for that accumulation. Fifteen individual typical nodes per z-series in normal subjects and 9–15 typical nodes in lesioned subjects were evaluated. The average intensity of paranodal caspr staining was determined from an average intensity z-projection of the caspr-alone z-series and included measurements of caspr staining on both sides of 7–17 typical nodes that were randomly selected from each z-series (53 nodes were each evaluated in lesioned IONs and normal IONs). The area of caspr staining on each side of the node where intensity was measured was identified as a "region of interest" or "ROI" in a merged maximum-intensity z-projection of the matching Na_v_1.6 combined with caspr z-series. This "ROI" was then added to the "ROI Manager" function found under the "Analyze" menu and applied to the caspr-alone average-intensity z-projection. A histogram was then obtained of this "ROI" that recorded the number of pixels, average intensity, and standard deviation of intensity staining within the area containing caspr staining. The nodal gap axial length (the gap between caspr) was measured at high magnification in these same nodes with the use of the "straight line selections" tool in the merged maximum-intensity z-projection. Since the number of individual slices within a z-series varied from 39–49, the Na_v_1.6 and caspr pixel value intensities obtained from each average-intensity z-projection were normalized to the z-projection with the greatest number of slices. All evaluations were done by one investigator to minimize variability in assessment.

### Statistical analysis

Statistical analysis to determine significance used the unpaired Student *t*-test while error bars on all graphs represent the standard error of the mean.

## Abbreviations

sodium channels (NaChs); infraorbital nerve (ION); region of interest (ROI); standard error of the mean (SEM).

## Competing interests

The author(s) declare that they have no competing interests.

## Authors' contributions

MH conceived the study, participated in its design, assisted with surgery, tissue preparation and drafted the manuscript. AF assisted with surgeries, tissue preparation and staining, collection of data, data analysis, and helped draft the manuscript. LJ participated in study design and performed the surgeries. SL helped to conceive the study, participated in its design, produced the Na_v_1.6 antibody, guided data analysis and helped draft the manuscript. All authors read and approved the final manuscript.
